# Inactivation and Unfolding of Protein Tyrosine Phosphatase from *Thermus thermophilus* HB27 during Urea and Guanidine Hydrochloride Denaturation

**DOI:** 10.1371/journal.pone.0107932

**Published:** 2014-09-25

**Authors:** Yejing Wang, Huawei He, Lina Liu, Chunyan Gao, Shui Xu, Ping Zhao, Qingyou Xia

**Affiliations:** 1 State Key Laboratory of Silkworm Genome Biology, Southwest University, Beibei, Chongqing, China; 2 College of Biotechnology, Southwest University, Beibei, Chongqing, China; Russian Academy of Sciences, Institute for Biological Instrumentation, Russian Federation

## Abstract

The effects of urea and guanidine hydrochloride (GdnHCl) on the activity, conformation and unfolding process of protein tyrosine phosphatase (PTPase), a thermostable low molecular weight protein from *Thermus thermophilus* HB27, have been studied. Enzymatic activity assays showed both urea and GdnHCl resulted in the inactivation of PTPase in a concentration and time-dependent manner. Inactivation kinetics analysis suggested that the inactivation of PTPase induced by urea and GdnHCl were both monophasic and reversible processes, and the effects of urea and GdnHCl on PTPase were similar to that of mixed-type reversible inhibitors. Far-ultraviolet (UV) circular dichroism (CD), Tryptophan and 1-anilinonaphthalene -8-sulfonic acid (ANS) fluorescence spectral analyses indicated the existence of a partially active and an inactive molten globule-like intermediate during the unfolding processes induced by urea and GdnHCl, respectively. Based on the sequence alignment and the homolog Tt1001 protein structure, we discussed the possible conformational transitions of PTPase induced by urea and GdnHCl and compared the conformations of these unfolding intermediates with the transient states in bovine PTPase and its complex structures in detail. Our results may be able to provide some valuable clues to reveal the relationship between the structure and enzymatic activity, and the unfolding pathway and mechanism of PTPase.

## Introduction

Although protein folding and unfolding have been extensively studied for several decades, it still attracts numerous researchers’ attention nowadays. Unfolding of small compact proteins is well defined as a simple two-state cooperative transition, in which only folded (native) and unfolded (denatured) molecules are populated at equilibrium [1], [2]. However, it is currently accepted that the unfolding/refolding of some proteins involve multiple processes. Some non-native states (such as molten globule state) with specific spectroscopic properties distinct from those of native and completely unfolded states have been observed under mildly denaturing conditions [3]–[5]. These conformational states are widely present and result in protein non-cooperative unfolding transitions. Characterizations of protein folding intermediates are important in identifying and understanding protein folding pathway and mechanism.

Protein tyrosine phosphorylation is of critical importance in the regulation of cell proliferation, differentiation and migration, the immune response and cytoskeletal reorganization [6], [7]. Reversible phosphorylation is controlled by a dynamic balance of opposing activities of protein tyrosine kinases (PTKase, EC 2.7.10.2) and protein tyrosine phosphatases (PTPase, EC 3.1.3.48) [8]. PTKases catalyze tyrosine’s phosphorylation with ATP as the substrate whereas PTPases catalyze the removal of phosphate from tyrosine residue [9]. PTPases belong to a large and structurally diverse family of enzymes, which specifically regulate a wide range of signaling pathways [10]. Lots of PTPases structures have been resolved to understand its substrate specificity, catalytic mechanism and biologic functions *in* *vivo* since the first purification of PTPase in 1988 [11]. Based on the structures and substrate specificities, the PTPase superfamily can be divided into four subfamilies: 1) classical pTyr specific PTPase, 2) dual specificity phosphatases, 3) Cdc25 phosphatases, and 4) low molecular weight (LMW) PTPase [12]. The structures of LMW PTPase are highly conserved from prokaryotic to eukaryotic organisms, which share a common PTPase signature motif or P-loop C(X)_5_R(S/T) located around the active sites [13], [14]. Defective or inappropriate PTPase activities will lead to a variety of diseases, including type II diabetes, cancer, dysfunctions of the immune system and infection by pathogenic bacteria [15], [16]. A number of PTPases have been taken into account to be strategic therapeutic targets such as diabetes and cancer due to their essential biological functions [17], [18]. Therefore, understanding the relationship between PTPase structure, enzymatic activity, folding mechanism and their functions *in* *vivo* is critical to better utilize PTPases as therapeutic targets for human diseases.

More and more attentions have been paid to an extremely thermophilic bacterium *Thermus thermophilus* to explore its potential scientific and economic value since the completion of the *Thermus thermophiles* genome project [19]. The crystal structure of Tt1001 protein from *Thermus thermophilus* HB8 (PDB ID: 2CWD), a classical LMW PTPase, has been resolved (Lokanath, N.K., Terao, Y., Kunishima, N. (2005), Crystal structure of Tt1001 protein from *Thermus Thermophilus* Hb8, unpublished.), however the protein’s enzymatic properties and its functions *in* *vivo* are still unknown. Our previous study has shown another PTPase from *Thermus thermophilus* HB27, a homolog of Tt1001, exhibits significant structural thermostability and high levels of residual activity treated under high temperature for half an hour [20]. However, at present, how the PTPase structure affects protein folding/unfolding states and its enzymatic activity is not yet fully understood. In this research, we studied the inactivation kinetics and unfolding processes of PTPase in the presence of urea and GdnHCl to explore the effects of these denaturants on the activity, secondary/tertiary structure and unfolding state of PTPase.

## Materials and Methods

### 1. Materials

Para-nitrophenyl phosphate (*p*NPP) was purchased from Amresco (USA). Urea, GdnHCl (ultrapure), DTT and 1-anilinonaphtalene-8-sulphonate (ANS) were products of Sigma (St. Louis, MO, USA). All other reagents were local analytical grade products.

### 2. Protein expression and purification

The gene encoding PTPase of *Thermus thermophilus* HB27 (Gene ID: 2775219) was successfully cloned and efficiently expressed in *Escherichia coli* BL21 [DE3]. PTPase was further purified as previously described [20]. The protein was purified and analysed to be homogeneous on 15% SDS-PAGE. The enzyme concentration was determined by BCA protein assay kit (Pierce, USA).

All experiments were generally performed in 50 mM sodium acetate buffer (pH 3.8) with 5 mM DTT. PTPase was incubated in the absence and presence of urea and GdnHCl for 3 h at 25°C before all the measurements were performed so that equilibrium was achieved. The final PTPase concentration was 2.4 µM for most experiments, unless mentioned specifically.

### 3. PTPase assay

The enzymatic activity was determined as described previously with minor modification [21]: the assay was carried out at 30°C in 200 µl reaction mixtures in the absence and presence of urea or GdnHCl. The reaction was terminated after incubation at 30°C for 10 min by addition of 1 ml 1 M NaOH. The changes in absorbance at 405 nm were recorded on a Helios γ spectrophotometer (Thermo spectronic, USA). The molar extinction coefficient of 1.80×10^−4^ M^−1^•cm^−1^ was used to calculate the amount of product in this reaction.

### 4. Kinetic analysis

**Figure 1:**
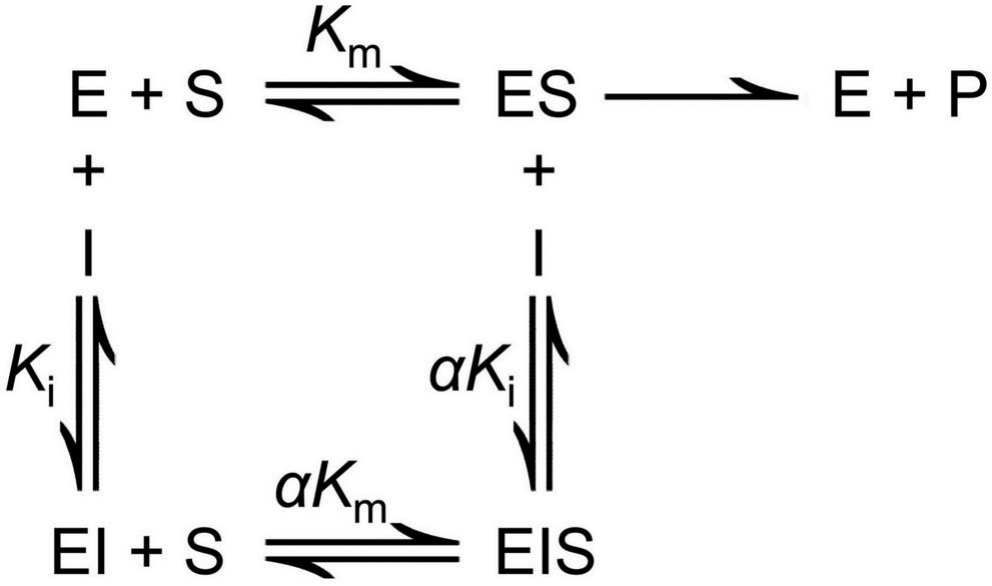
The illustration of mixed-type inhibition mechanism. E, S, I and P represent enzyme, substrate, inhibitor and product, respectively.



For the analysis of a mixed-type inhibition mechanism (Fig. 1) [22], the Lineweaver-Burk equation in the double reciprocal form can be written as:

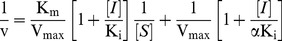
(1)The following equation (2) and (3) can be deduced based on the equation (1),
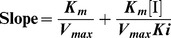
(2)

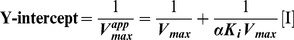
(3)


The plot of *slope versus* [I] and *y-intercept versus* [I] converts the relationship to a straight line so that the values of *K_i_* and *α* can be calculated according to the above equations.

### 5. Fluorescence spectroscopy

The fluorescence emission spectra were recorded at 25°C on a F-2500 fluorescence spectrophotometer (Hitachi, Japan). Intrinsic fluorescence was recorded in the wavelength range 300–400 nm after exciting at 280 nm with a 1 cm pathlength quartz cuvette. PTPase was incubated in the presence of urea or GdnHCl for 3 h at 25°C before the spectra were recorded. All fluorescence spectra were corrected by subtraction of the apparent fluorescence of the respective concentrations of urea or GdnHCl under the same conditions. The final spectrum was an average of three corrected spectra.

For ANS fluorescence measurements, 120 µM ANS were added into PTPase solutions in the presence of urea or GdnHCl to react for 30 min in the dark before the spectra were recorded. ANS fluorescence emission spectra were collected in the wavelength range 400–600 nm with an excitation wavelength 380 nm. The final spectrum was an average of three scans after subtraction of the buffer containing the appropriate concentration of denaturants.

### 6. CD spectroscopy

Far-UV circular dichroism (CD) spectra were performed at 25°C on a Jasco J-715 spectrophotometer (Jasco, Japan). The spectra were recorded over a wavelength range 200–250 nm using a 2 mm pathlength quartz cuvette. The final concentration of PTPase was 11 µM. Each spectrum was an average of five scans. The spectra were corrected by subtracting the baseline recorded for the buffer containing the respective concentration of denaturants under the same conditions.

### 7. Protein sequence alignment and crystal structure visualization

The amino acid sequence of PTPase (Pubmed ID: YP_004789.1) was used to search its homolog proteins structures in protein database bank (PDB). Tt1001 protein from *Thermus thermophilus* HB8 shows 100% sequence identity with PTPase and its crystal structure has been resolved at 1.90 Å resolution. The sequence alignment of PTPase with Tt1001 protein was completed by Clustal X [23] and rendered by ESPript 3 with 2CWD as the secondary structural template [24]. The Tt1001 protein crystal structure was visualized by Pymol (The PyMOL Molecular Graphics System, Version 0.99, Schrödinger, LLC.).

## Results

### 1. Effects of urea and GdnHCl on the activity of PTPase

**Figure 2:**
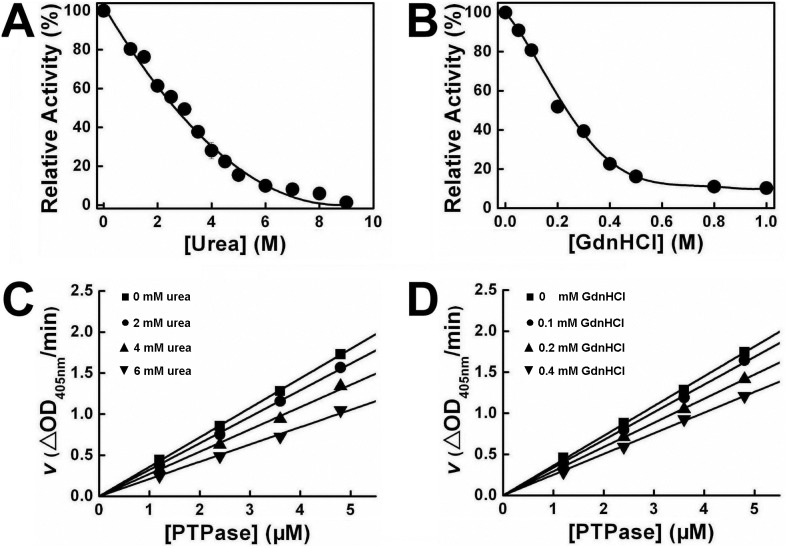
Inactivation of PTPase induced by urea (A) and GdnHCl (B). The plots of enzymatic activity *versus* [*PTPase*] in the presence of different concentrations of urea (C) and GdnHCl (D).

To explore the effects of urea and GdnHCl on the enzymatic activity of PTPase, the relative residual activities of PTPase in the presence of different concentrations of urea and GdnHCl were measured, as presented in Fig. 2A&B, respectively. PTPase activity decreased gradually with increasing urea and GdnHCl concentrations, as shown in Fig. 2A&B. The values of IC_50_, defined as the denaturant concentration required for 50% activity inhibition, were expected to be 2.65 M for urea and 0.24 M for GdnHCl, respectively. PTPase activity was almost completely lost in 9 M urea or 1 M GdnHCl, indicating that the conformation of the active sites of PTPase almost have been completely changed by these denaturants. The plots of PTPase residual activity in urea or GdnHCl as a function of PTPase concentration showed a series of straight lines which all pass through the origin, as shown in Fig. 2C&D, respectively. The slopes of these lines decreased with increasing urea and GdnHCl concentrations, indicating that urea and GdnHCl were both reversible denaturants of PTPase.

**Figure 3:**
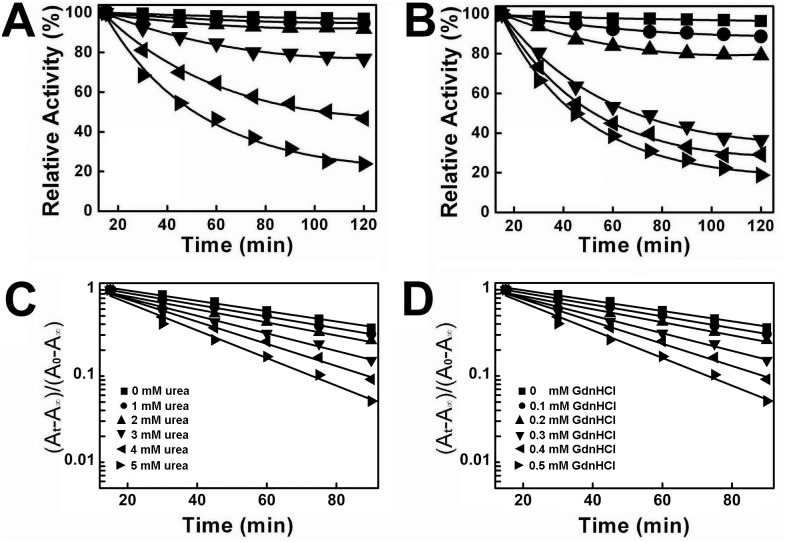
Inactivation kinetics of PTPase in the presence of different concentrations of urea (A) and GdnHCl (B). (C–D) Semilogarithmic plots for urea and GdnHCl, respectively.

The kinetics of PTPase inactivated by urea and GdnHCl were studied to reveal the inactivation kinetic mechanism including inactivation rate constants, reactive type and other kinetic parameters. Fig. 3A&B showed the inactivation kinetics of PTPase in the presence of a series concentrations of urea and GdnHCl, respectively. The results showed that the PTPase activity was gradually lost in a time-dependent manner in the presence of urea or GdnHCl. Semi-logarithmic plots (Fig. 3C&D) indicated the inactivation of PTPase induced by either urea or GdnHCl was a typical kinetic monophasic process. The apparent kinetic constants of PTPase inactivated by urea and GdnHCl were calculated and presented in Table 1 and 2, respectively.

**Table 1:** The inactivation rate constants and residual activity of PTPase in the presence of different concentrations of urea.

Urea (M)	Inactivation rate constant *A* (×10^−3^s^−1^)	Residual activity (%)
0	0	100.00
1	0.634	80.39±0.63
2	0.501	61.35±0.78
3	0.558	49.41±0.21
4	0.474	28.04±2.24
5	0.489	15.44±1.12

**Table 2:** The inactivation rate constants and residual activity of PTPase in the presence of different concentrations of GdnHCl.

GdnHCl (M)	Inactivation rate constants *A* (×10^−3^s^−1^)	Residual activity (%)
0	0	100.00
0.1	0.432	80.76±0.35
0.2	0.686	51.87±1.20
0.4	0.647	22.65±0.85
0.6	0.619	12.70±0.68
0.8	0.555	10.99±0.78

**Figure 4:**
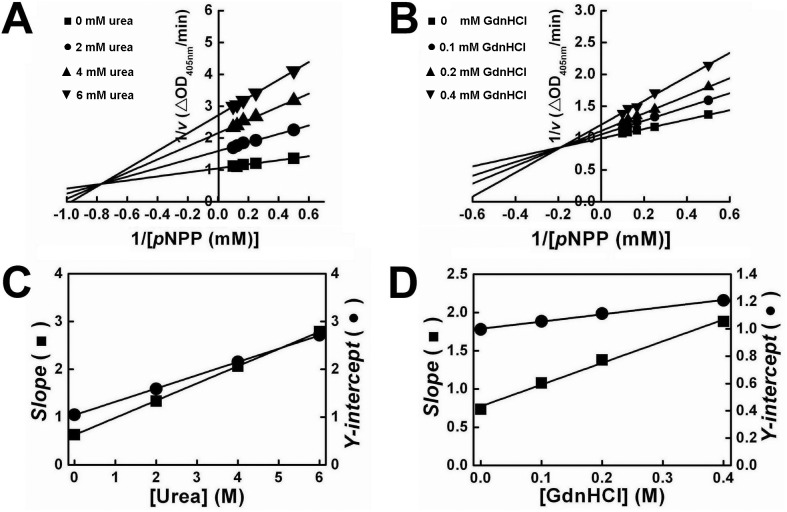
Inhibition kinetics of PTPase in the presence of different concentrations of urea and GdnHCl. Lineweaver-Burk plots for urea (A) and GdnHCl (B). (C–D) The secondary plots of slope and y-intercept *versus* [urea] and [GdnHCl], respectively.

Fig. 4A&B showed the Lineweaver-Burk plots of PTPase in the presence of a series concentrations of urea and GdnHCl, respectively. Both of these plots were intersected at the second quadrant and the apparent *K*_m_ values increased along with the decrease of the apparent *V*_max_ values, indicating the effects of urea and GdnHCl on the inactivation kinetics of PTPase resembled that of a mixed-type inhibitor. The secondary plots of *slope* and *y-intercept vs* urea or GdnHCl concentrations showed as a straight line (Fig. 4C&D), which was in good agreement with the previously proposed model of mixed-type inhibition. The values of *K*_i_ and α for the two denaturants were calculated and presented in Table 3. Either IC_50_ or *K*_i_, the value of GdnHCl was much smaller than that of urea, indicating that GdnHCl was a much more effective denaturant than urea for PTPase.

**Table 3:** The inhibition and inactivation kinetic parameters of PTPase in the presence of urea and GdnHCl.

Denaturants	Inhibition type	IC*_50_*	*K*_i_	α
Urea	Mixed	2.65±1.21 M	1.74 M	2.16
GdnHCl	Mixed	240±1.01 mM	0.27 mM	6.99

### 2. Urea and GdnHCl induced intrinsic fluorescence spectra changes of PTPase

**Figure 5:**
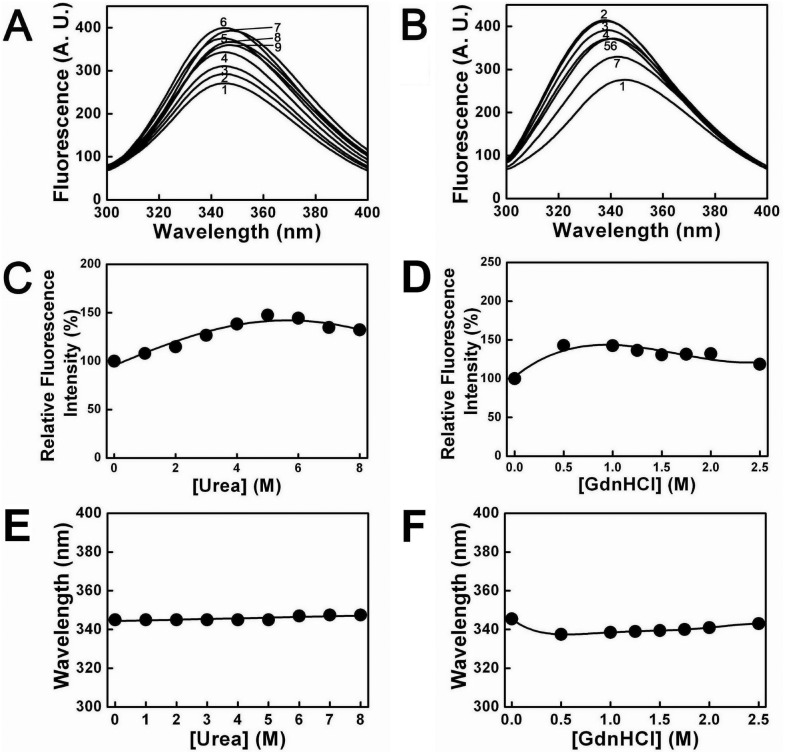
Intrinsic fluorescence spectra of PTPase in the presence of different concentrations of urea and GdnHCl. (A) Urea concentrations for the labels 1–9 were 0, 1, 2, 3, 4, 5, 6, 7 and 8 M, respectively. (B) GdnHCl concentrations for the labels 1–7 were 0, 0.5, 1, 1.25, 1.5, 2 and 2.5 M, respectively. (C–D) The relative changes of Imax value as a function of [urea] and [GdnHCl], respectively. (E–F) The relative changes of λmax value as a function of [urea] and [GdnHCl], respectively.

The intrinsic fluorescence emission spectra of PTPase in the presence of urea and GdnHCl were recorded from 300 nm to 400 nm to monitor the conformational changes around the Trp residues of PTPase during the unfolding processes induced by urea and GdnHCl. Fig. 5A&B showed the intrinsic fluorescence spectra changes of PTPase in the presence of different concentrations of urea and GdnHCl, respectively. In 0–5 M urea, the maximum fluorescence emission intensity (Imax) of PTPase increased with increasing urea concentrations. Imax was about 1.5 fold in 5 M urea as compared to that of native protein. While further increasing urea concentrations up to 8 M, Imax began to decrease to about 125% of native PTPase (Fig. 5C), and the maximum fluorescence emission wavelength (λmax) red-shifted from 345.5 nm to 347.5 nm (Fig. 5E), suggesting the conformation of PTPase had been gradually changed that the Trp residues was accessed by solvents more easily duo to the unfolding of PTPase induced by urea.

As shown in Fig. 5D, Imax first increased and then decreased with increasing GdnHCl concentrations. In 0.5 M GdnHCl, Imax increased to about 150% of native protein. While further increasing GdnHCl concentrations to 2.5 M, Imax began to decline to about 120% of native PTPase. In contrast to Imax, λmax first blue-shifted and then red-shifted with increasing GdnHCl concentrations. While increasing GdnHCl concentrations to 0.5 M, λmax blue-shifted from 345.5 nm to 337.5 nm. With further increasing GdnHCl concentrations to 2.5 M, λmax subsequently red-shifted from 337.5 nm to 343 nm (Fig. 5F).

### 3. Urea and GdnHCl induced ANS fluorescence spectra changes of PTPase

**Figure 6:**
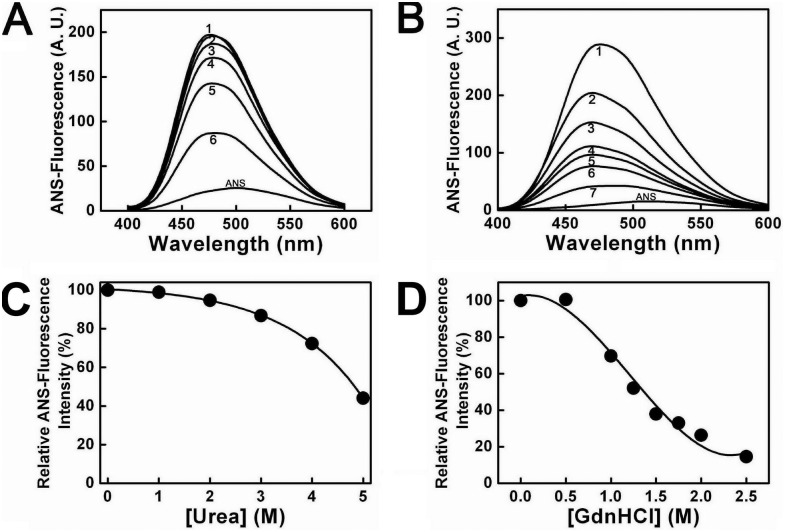
ANS binding fluorescence spectra of PTPase in the presence of different concentrations of urea and GdnHCl. (A) Urea concentrations for the labels 1–6 were 0, 1, 2, 3, 4 and 5 M, respectively. (B) GdnHCl concentrations for the labels 1–7 were 0, 1.00, 1.25, 1.50, 1.75, 2.00 and 2.50 M, respectively. (C–D) The relative changes of Imax value as a function of [urea] and [GdnHCl], respectively.

Further, to probe the exposure of hydrophobic residues, which were buried in the folded state of PTPase, the extrinsic ANS fluorescence emission spectra were conducted from 400 nm to 600 nm. The effects of urea and GdnHCl on the ANS fluorescence spectra of PTPase were shown in Fig. 6A&B, respectively. Once binding with native PTPase, λmax of ANS fluorescence blue-shifted from about 500 nm to 475 nm. With increasing urea concentrations to 5 M, the Imax value decreased significantly, while λmax virtually did not vary, indicating the ANS-binding buried hydrophobic patches of PTPase had been exposed to solvents gradually. In 5 M urea, Imax declined to about 45% of native PTPase, as shown in Fig. 6C.

In the presence of GdnHCl, λmax of ANS fluorescence blue-shifted from about 500 nm to 470 nm. Distinct from that of urea, Imax almost did not vary in 0–0.5 M GdnHCl. While further increasing GdnHCl concentrations up to 2.5 M, Imax significantly decreased to about 10% of native PTPase, while λmax almost did not change (Fig. 6D), suggesting the buried hydrophobic patches of PTPase almost had been exposed to solvents completely.

### 4. Urea and GdnHCl induced Far-UV CD spectra changes of PTPase

**Figure 7:**
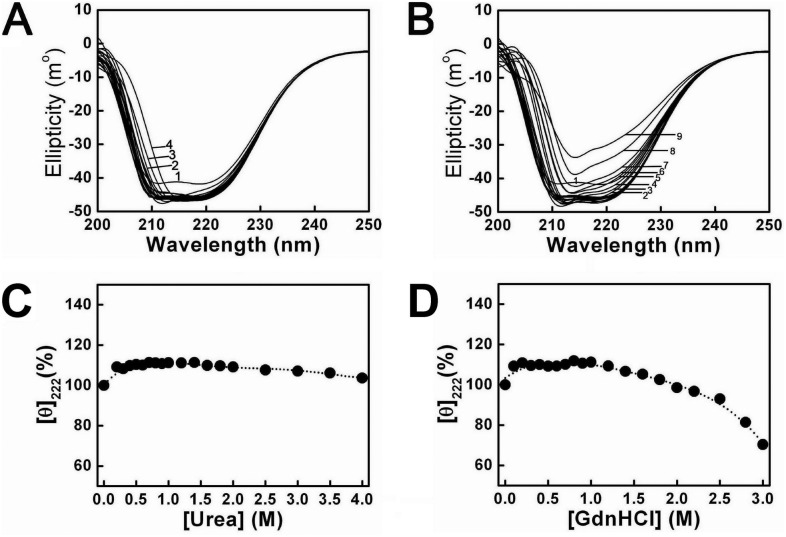
Far-UV CD spectra of PTPase in the presence of different concentrations of urea and GdnHCl. (A) Urea concentrations for the labels 1–4 were 0, 3, 3.5 and 4 M, respectively. (B) GdnHCl concentrations for labels 1–9 were 0, 1.4, 1.6, 1.8, 2, 2.2, 2.5, 2.8 and 3 M, respectively. (C–D) The relative changes of θ_222_ as a function of [urea] and [GdnHCl], respectively.

Far-ultraviolet circular dichroism (CD) spectra were measured from 200 nm to 250 nm to monitor the α-helix structural transitions of PTPase induced by urea and GdnHCl. Fig. 7A&B showed the far-UV CD spectra changes of PTPase in the presence of different concentrations of urea and GdnHCl, respectively. The relative ellipticity values at 222 nm (θ_222_) in far-UV CD spectra, a typical signal of protein’s α-helix structure, *vs* urea or GdnHCl concentrations were shown in Fig. 7C&D, respectively. Here the θ_222_ value of native protein was considered to be 100%. The θ_222_ value first increased and then declined slightly with increasing urea concentrations from 0 to 4 M (Fig. 7C). In the presence of 0.2 M urea, the θ_222_ value increased to about 109% as compared to that of native PTPase, suggesting that 0.2 M urea induced other secondary structures such as β-sheets, β-turns or random coils of PTPase to transform into α-helix structures, thus resulted in the α-helix structural contents increase. While increasing urea concentration to 2 M, the θ_222_ value virtually did not change, indicating the α-helix contents of PTPase did not change. With further increasing urea concentrations more than 2 M, the θ_222_ value began to decline gradually, indicating the α-helix structures were induced to unfold or transform into other secondary structures. In 4 M urea, the θ_222_ value decreased to about 103% of native protein (Fig. 7C). In a word, the θ_222_ values in different concentrations of urea reflected the changes of the α-helix structural contents of PTPase.

The α-helix structural changes of PTPase induced by GdnHCl resembled that by urea, as shown in Fig. 7D. In 0.2 M GdnHCl, the θ_222_ value increased to about 109% compared to that of native PTPase, suggesting that 0.2 M GdnHCl also was able to induce the increase of the α-helix structures of PTPase. While increasing GdnHCl concentrations to about 1.0 M, the θ_222_ value almost did not change, indicating the α-helix structural contents of PTPase were not affected by increasing GdnHCl concentrations. Distinct from urea, the θ_222_ value began to decline when increasing GdnHCl concentrations more than 1 M. In 2 M GdnHCl, the θ_222_ value decreased to about 98% of native PTPase. While further increasing GdnHCl concentrations to 3 M, the θ_222_ value decreased to about 70% of native protein, suggesting about 30% α-helix structures of PTPase had been induced to unfold or transform into other secondary structures.

### 5. Sequence alignment and Tt1001 protein structural analysis

**Figure 8:**
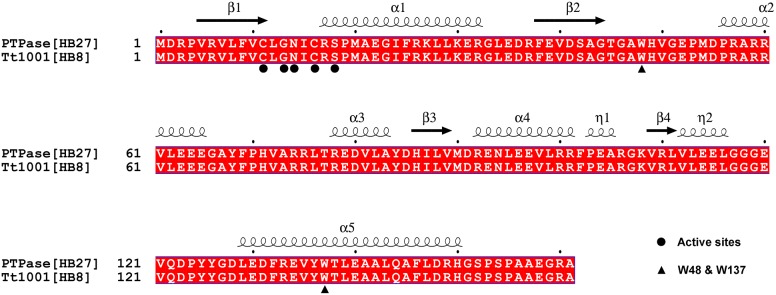
The sequence alignment of PTPase with Tt1001 protein.

To reveal the relationship between the structure of PTPase and its activity and unfolding conformational state induced by urea and GdnHCl, the amino acid sequence of PTPase was used to search its homolog protein structure in PDB. Fortunately, about 38 homolog proteins structures which show certain sequence identities with PTPase have been resolved. Among of them, Tt1001 protein from *Thermus thermophilus* HB8 shows 100% sequence identity with PTPase (Fig. 8), suggesting the structure of Tt1001 protein is closely similar to that of PTPase.

**Figure 9:**
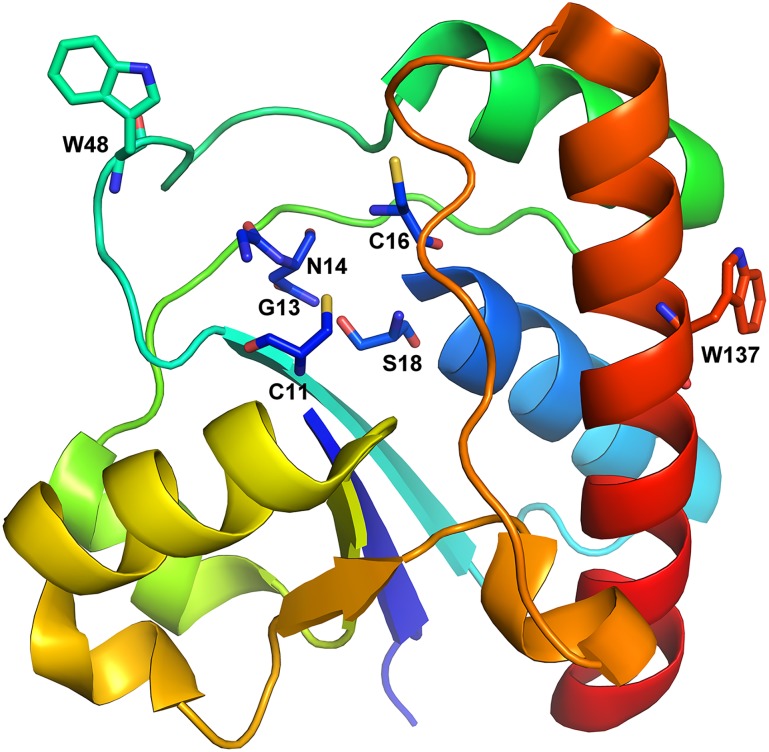
Cartoon representation of the one molecule in the asymmetric unit of the Tt1001 crystal structure. The active sites Cys11, Gly13, Asn14, Cys16 and Ser18 of Tt1001 and Trp48, Trp137 are represented as sticks.

The 1.90 Å crystal structure of Tt1001 protein reveals two molecules forming a dimer in the asymmetric unit of the crystal. Each monomer contains a CX_5_R(S/T) signature motif or P-loop which is essential to the phosphate binding and catalytic activity. The structure shows that Cys11, Gly13, Asn14, Cys16 and Ser18 located in the P-loop form a relatively closed cleft which serves as the active center. The only two tryptophan residues, W48 and W137 both expose on the surfaces of PTPase (Fig. 9), which are readily accessible to solvents and in good agreement with the λmax value 345.5 nm of the intrinsic fluorescence of native PTPase. W48 locates on a flexible loop close to the active center, and W137 locates on a long α-helix (α5) structure which is far away from the active center. Compared to the α5-helix structure, the conformation of the flexible loop including W48 residue may be changed by denaturants more easily, which thus affect the conformation of the adjacent active sites and the activity of PTPase.

## Discussion

The denaturation of small protein is generally believed to be a highly cooperative process, which may be approximated by a two-state model and no significant intermediates are present during the transition from a native state (N) to a denatured state (D) [25], [26]. However, recent results show the existence of intermediates between native and unfolded states, which are found in mildly denaturing conditions and referred as molten globules in some cases [27]–[31]. The peptide chains in the molten globule state are nearly as compact as that in the native state [31], and usually characterized for the existence of pronounced secondary structures by far-UV CD spectra [31]–[33].

Urea and GdnHCl are the most common chemical denaturants, which are usually used to denature proteins and characterize the conformation, stability, and folding/unfolding pathway and mechanism of proteins [34]–[37]. Urea and GdnHCl have shown a variety of behaviors towards different proteins [37]–[42]. For example, low concentrations of urea or GdnHCl are able to increase the enzymatic activity of prostaglandin d-Synthase [41]. As for matrilysin, half of the fluorescence has been changed in 2.2–2.7 M GdnHCl, whereas nothing has happened even in 8 M urea [42]. Here we studied the effects of urea and GdnHCl on the activity, conformation and unfolding state of PTPase from *Thermus thermophilus* HB27.

The activity assay and inactivation kinetics suggest the inactivation of PTPase induced by urea or GdnHCl is a monophasic, time and concentration-dependent reversible process. Also the effects of urea and GdnHCl on PTPase activity are similar to that of mixed-type inhibitors. In addition, GdnHCl has shown to be much more effective than urea to induce the inactivation and unfolding of PTPase, which is in accordance with the results of the intrinsic and ANS fluorescence as well as CD spectra.

The α-helix structural contents of PTPase increased slightly in 0.2 M urea, then did not vary until about 2 M urea, while in 0.2–2 M urea, the Imax and λmax values of the intrinsic fluorescence changed a little, and the residual activity still remained more than 60% of native PTPase. These results together suggest that 0.2–2 M urea may induce a slight conformational change around the active sites of PTPase, which result in a partial activity loss, a slight red-shift of the intrinsic fluorescence λmax and a slight decrease of the ANS fluorescence Imax as well as the α-helix structural contents increase, indicating the existence of a partially active molten globule-like intermediate. The formation of a molten globule-like intermediate may result in the decrease of the Imax value of the intrinsic fluorescence due to the exposure of the Trp residues of PTPase. However, the Imax value of the intrinsic fluorescence increased gradually in 0–5 M urea, which may arise from the increase of solution viscosity due to increasing urea concentration.

The α-helix structural contents of PTPase increased to 109% of native protein in 0.2 M GdnHCl, then did not alter until about 1 M GdnHCl. While in 0.5 M GdnHCl, more than 80% activity of PTPase had been lost, indicating most of the conformations of the active sites had been changed. In addition, the Imax value of the intrinsic fluorescence increased to about 150% and λmax blue-shifted about 8 nm compared to that of native protein, clearly indicating a significant conformational change around the Trp residues of PTPase. These results together reveal that low concentrations GdnHCl (≤0.5 M) may induce a significant conformational change of the active sites and tertiary structures of PTPase, which result in the loss of the activity, promote the formation of α-helix structure and an inactive molten globule-like intermediate.

The formation of a partially active/inactive intermediate could be due to the conformational changes around the Trp residues and the active sites, small local structural rearrangements of native state [40] or the stabilizing effects of Gdn^+^ on protein conformation at low concentrations [43], [44]. Previous studies have shown the different effects of GdnHCl and urea on the conformational stability of protein [41], [42], [45]–[48]. While further increasing denaturants concentrations more than 2 M urea or 0.5 M GdnHCl, the conformation of the partially active/inactive intermediate was induced continually by denaturants to bring about the exposure of the hydrophobic patches and the unfolding of PTPase, which finally result in the decrease of the Imax value of the intrinsic and ANS fluorescence and λmax’s red-shift as well as the complete loss of activity and the decrease of α-helix structural contents.

The difference in the activity of molten globule-like intermediate present in GdnHCl and urea also suggest that GdnHCl is much more effective than urea when used to inactive PTPase, which may arise from that GdnHCl could induce a significant conformational change of the active sites by the electrostatic interactions of Gdn^+^ with the charged groups around the active sites of PTPase [43], [44], [49], [50]. In fact, we did notice that the acidic Glu and Asp residues contents are up to 19.2%, and the basic Arg residues content is up to 12.4%. The Tt1001 protein structure suggests most of these charged residues such as E35, D36, R37, E39, D41, E52, D55, R57, R59 and R60 surround the active sites and expose to solvents. Once these residues interact with Gdn^+^ via the electrostatic interactions, the barrier which protects the conformation of the active sites will be destroyed quickly, which thus affect the conformation of the active sites and result in the activity loss of PTPase.

Although our previous gel filtration analysis indicated PTPase was a monomer in solution, which is different from the dimer state in the crystal structure of Tt1001, the Tt1001 protein structure may be still valuable to reveal the conformational transition of PTPase induced by different concentrations urea and GdnHCl. While comparing the location of W48 and W137, we may be able to conclude low concentrations urea (≤2 M) or GdnHCl (≤0.5 M) may first induce the conformational changes of the flexible loop including the easily accessible W48 residue rather than the α5 structure with W137 residue, thus result in the conformational changes of the adjacent active sites and the activity loss as well as the increase of α-helix structural contents. The difference between urea and GdnHCl is the conformational changes of the active sites induced by urea are not as obvious as that by GdnHCl, as the residual activity of PTPase in 2 M urea was still much higher than that in 0.5 M GdnHCl. The conformational change of the loop may result in the W48 residue is accessed by solvents more easily, as indicated by a slight red-shift of λmax. However, GdnHCl may induce a more significant conformational changes of the flexible loop which resulted in the W48 residue was buried into the interior of PTPase and not accessible to solvents easily, as observed an obvious 8 nm blue-shift of λmax and the increase of the Imax value. Also, this flexible loop was likely induced by GdnHCl to transform into α-helix structures, which resulted in the increase of α-helix structural contents. Although this inference based on the Tt1001 protein structure is consistent with our experimental observations, we are still not able to exclude the possibilities that in the low concentrations of urea (≤2 M) or GdnHCl (≤0.5 M), the conformational changes of other loops or secondary structures may contribute to the activity loss and the increase of α-helix structural contents, and the conformational changes around W137 residue probably cause the intrinsic and ANS fluorescence spectra changes as well as the increase of α-helix structural contents, more evidences are needed to reveal the conformational changes in detail.

The crystal structure of bovine PTPase (bPTPase) (PDB ID: 1DG9) has been resolved at 1.90 Å [51], which shows about 41% sequence identities and shares a common signature motif C(X)_5_R(S/T) with PTPase. The structure of 1DG9 reveals a dimer formed by Tyr131 and Tyr132 from two different monomers at pH 7.0 in 0.1 M Tris. However, Tabernero et al also demonstrate that the native bPTPase and S19A mutant exist as a monomer at pH 4.8 in the low ionic strength buffer, which is consistent with our previous gel-filtration analysis that PTPase existed as a monomer in 50 mM, pH 3.8 sodium acetate buffer. In addition, the dimer structure of bPTPase may represent a transient state between an inactive and active state. The activity of bPTPase seems to be dependent on the phosphorylation or dephosphorylation of tyrosine, which will affect the conformation of the variable loop and thus result in the opening or closing of the active sites. The structures of bPTPase complexed with its inhibitors vanadate and molybdate at 2.2 Å resolution (PDB ID: 1Z12 and 1Z13) also reveal a reactive transition state in the reaction catalyzed by PTPase [52]. The conformation of the partially active/inactive intermediates present in low concentrations of urea and GdnHCl seems to be similar to these transition states as these denaturants was also able to induce the conformational changes of the variable loop thus affect the activity of enzyme, however, no obvious conformational changes are observed in the structures of bPTPase and its complexes, which is different from the conformations of these intermediates in this research, as at least a slight increase of α-helix structure was found to exist in these unfolding intermediates of PTPase.

**Figure 10:**
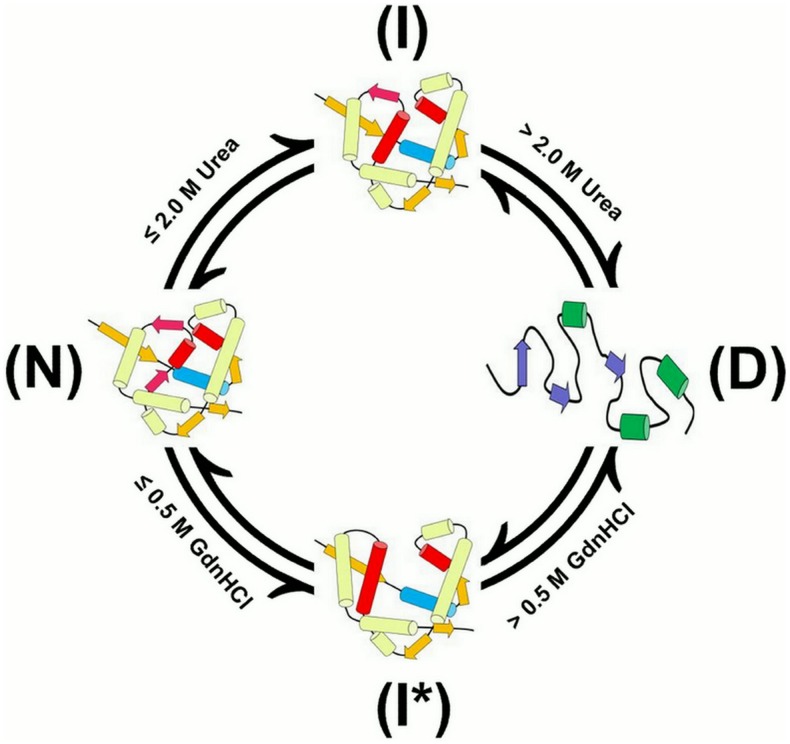
The different unfolding intermediates of PTPase in the presence of urea and GdnHCl. N, I, I* and D represent native state, partially active intermediate state, inactive intermediate state and denatured state, respectively.

In conclusion, our results reveal the existence of different unfolding intermediates during the unfolding processes of PTPase induced by urea and GdnHCl (Fig. 10). Although the specific unfolding pathway and mechanism still remain unclear, at least our research could provide some experimental evidences about the different effects of urea and GdnHCl on the conformation and activity of PTPase, which may be valuable to reveal the relationship between PTPase structure and its activity, as well as the protein folding pathway and mechanism in the future.
